# Effect of High-dose Vitamin D on IL-1β Blood Level in Patients with Moderate Stroke: A Randomized Clinical Trial

**DOI:** 10.5812/aapm-138810

**Published:** 2023-08-19

**Authors:** Mehran Kouchek, Seyedpouzhia Shojaei, Saied Amniati, Mehran Ghaffari, Sara Salarian, Mir Mohammad Miri, Niloufar Taherpour, Farnoosh Masbough, Mohammad Sistanizad

**Affiliations:** 1 Critical Care Quality Improvement Research Center, Shahid Beheshti University of Medical Sciences, Tehran, Iran; 2 Department of Anesthesia and Critical Care Medicine, Imam Hossein Medical Center, Shahid Beheshti University of Medical Sciences, Tehran, Iran; 3 Department of Anesthesia and Critical Care Medicine, Critical Care Quality Improvement Research Center, Shohada-e Tajrish Hospital, Shahid Beheshti University of Medical Sciences, Tehran, Iran; 4 Department of Anesthesia and Critical Care Medicine, School of Medicine, Shahid Beheshti University of Medical Sciences, Tehran, Iran; 5 Department of Neurology, Shahid Beheshti University of Medical Sciences, Tehran, Iran; 6 Prevention of Cardiovascular Disease Research Center, Imam Hossein Medical Center, Shahid Beheshti University of Medical Sciences, Tehran, Iran; 7 Department of Clinical Pharmacy, Faculty of Pharmacy, Shahid Beheshti University of Medical Sciences, Tehran, Iran

**Keywords:** Vitamin D, IL-β, Stroke, Interleukin, ICU

## Abstract

**Background:**

Vitamin D has neuroprotective and anti-inflammatory effects in stroke patients, but its effect on pro-inflammatory and inflammatory cytokines, especially IL-1, has been investigated in a few trials.

**Objectives:**

This study aimed to determine the effect of prescribing a high dose of vitamin D on the anti-inflammatory parameters, short-term and long-term prognosis of patients with ischemic stroke.

**Methods:**

This randomized clinical trial was performed on 42 patients randomly divided into two equal groups of 21 in Imam Hussein Hospital. The patients were allocated through block randomization methods to receive 300,000 units of vitamin D (intramuscularly) or not receive it as a control group. Age, gender, and clinical and laboratory information were recorded. The stroke severity was calculated according to the National Institute of Health Stroke Scale (NIHSS) at the beginning of hospitalization and upon hospital discharge. The 3-month prognosis of the patients was recorded according to the Barthel criteria three months after the stroke. Vitamin D3 levels were recorded before and after injection, while the neutrophil-to-lymphocyte ratio (NLR) and platelet-to-lymphocyte ratio (PLR) were assessed on the first day and for 7 consecutive days after hospitalization. All statistical analyses were performed using STATA version 14. A P-value < 0.05 was considered significant.

**Results:**

The mean age of the patients was 61.45 ± 4.74 years. There were 18 female (42.86%) and 24 male patients (57.14%). In the vitamin D group, the mean IL-1 decreased compared to before the intervention (-23.60 ± 103.83), but this decrease was not statistically significant (P = 0.070). In addition, the changes in IL-1 after the intervention were statistically different between the two groups (mean difference of -23.60 ± 103.83 in the vitamin D group vs. 15.96 ± 9.64 in the control group). The mean IL-6 decreased in both groups after the intervention compared to before, although these changes were not statistically significant (P > 0.05). In the group receiving vitamin D compared to the control group, the mean NLR decreased by about 2 units, the PLR decreased by about 10 units, and the NIHSS score decreased by about one unit during the study. However, these changes were not statistically significant (P > 0.05).

**Conclusions:**

A high dose of vitamin D can improve the NIHSS score and decrease IL-1 and IL-6, although these changes were not statistically significant. The mean NLR and PLR decreased after using high-dose vitamin D.

## 1. Background

Stroke is a major public health problem, and intensive rehabilitation is an important part of recovery after stroke (1). Approximately 16 million strokes and 5.7 million stroke-related deaths occur annually worldwide (2). Stroke is one of the main causes of chronic physical disorders that impacts a person's ability to perform daily activities. Functional recovery after stroke remains a high priority in health care (3). Data analysis from the Global Burden of Disease 2019 study showed that from 1990 to 2019, the absolute number of strokes increased by 70% (4).

Previous research has shown that 86% to 89% of elderly patients in a rehabilitation center had vitamin D deficiency, and several other studies have reported an association between biomarkers like serum vitamin D levels and functional improvement after stroke (5-9). Vitamin D is a neurosteroid thought to play a neuroprotective role due to the widespread distribution of vitamin D receptors on neurons and glial cells (10-12). The European Society of Clinical Nutrition and Metabolism recommends regular monitoring of the need for vitamin D supplementation and adequate dietary vitamin D intake for several neurological diseases (13).

Many inflammatory factors can be involved as acute or chronic stroke risk factors. In addition, inflammatory factors can affect the patient's prognosis after a stroke. Interleukin 1 is considered the most important factor in initiating inflammatory changes in the central nervous system. Recently, the inhibition of interleukin 1 has been proposed as an essential factor in controlling brain damage caused by traumatic brain events or strokes. Inhibition of interleukin 1 using interleukin 1 receptor blockers can be valuable in treating stroke (14). Interleukin 1 can be considered a therapeutic target in stroke. In the interleukin 1 family, about 11 members have been identified, most of which are interleukin 1 alpha and beta. The primary isoform of both mentioned interleukins is a 31-kDa protein, which later turns into a 17-kDa biologically active form. Although pro-IL-1α is slightly biologically active, pro-IL-1β is completely inactive. In brain strokes, due to the factors released from the damaged cells, the level of interleukin 1 increases, and the inactive form becomes active (15).

Clinical studies show that vitamin D treatment after a brain injury reduces cerebral edema, damage caused by free radicals, inflammatory cytokines TNF-α, IL-6, and nitric oxide (NO), and neurological disorders after ischemia (16). In addition to the blood level of interleukins, neutrophil-to-lymphocyte ratio (NLR) and platelet-to-lymphocyte ratio (PLR) are also considered inflammatory indicators in various diseases. The NLR can be used as a predictive factor for short-term and long-term prognosis in patients with stroke, and the increase of NLR is significantly and independently related to the severity of stroke (17, 18).

Platelets play an important role in regulating the immune system and inflammatory processes. An increase in platelet count can indicate a destructive inflammatory response and a prothrombotic state because some inflammatory mediators can stimulate the proliferation of megakaryocytes and thus lead to thrombocytosis. The PLR is a potential marker to determine the prognosis of inflammatory processes (19).

Despite the possible neuroprotective effects of vitamin D in stroke patients and its anti-inflammatory antioxidant effects (20), clinical trials are rare on the effect of this vitamin on pro-inflammatory and inflammatory cytokines, especially IL-1. Also, the dose and duration of its administration are unknown. In addition, the improvement of clinical outcomes in vitamin D supplementation in these patients has not been well revealed.

## 2. Objectives

Considering the high burden of stroke and the role of inflammatory factors in the prognosis of this disease, we aimed to determine the effect of prescribing a high dose of vitamin D on anti-inflammatory parameters in the short-term and long-term prognosis of patients with ischemic stroke.

## 3. Methods

**Figure 1.**
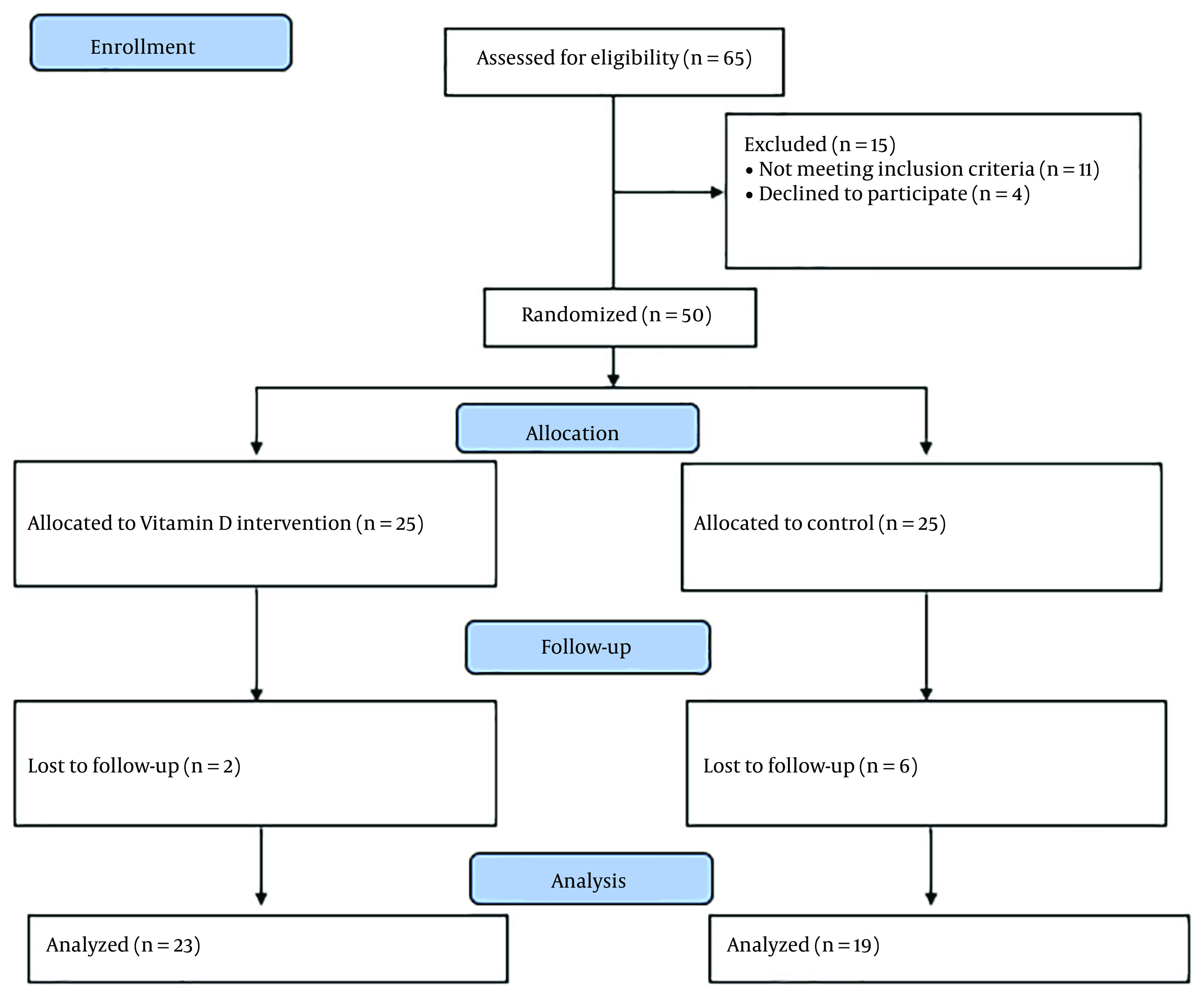
CONSORT flowchart

This open-labeled randomized clinical trial was performed on 42 patients randomly divided into two equal groups of 21 patients in Imam Hussein Hospital. The patients were allocated through block randomization to receive vitamin D or not receive it as a control group (Figure 1). Because no similar study was found on IL-β, a study with maximum similarity was used that investigated the effect of the Anakinra drug on IL-6 level in patients with subarachnoid hemorrhage following brain injury (21). Assuming the mean and standard deviation of IL-6 as 6.37 ± 4.25 in the intervention group and 11.04 ± 4.02 in the control group post-intervention, an alpha of 0.05, and a power of 90%, a sample of 17 people was calculated for each group. Finally, 21 people in each group were selected, assuming a dropout of 15%.

All hospitalized stroke patients were treated according to the latest relevant guidelines. Patients in the intervention group received one intramuscular injection of vitamin D3 with a dose of 300,000 units. We recorded demographic information of the patients, such as age and gender, and clinical and laboratory information. The stroke severity was calculated and recorded according to the National Institute of Health Stroke Scale (NIHSS) criteria at the beginning of hospitalization and upon discharge from the hospital. The 3-month prognosis of the patients was recorded based on the Barthel criteria 3 months after the stroke. Vitamin D3 levels were determined before and after injection, while the NLR and PLR were assessed on the first day and for 7 consecutive days after hospitalization. Blood samples were taken for IL-1β on the first day of hospitalization and 3 days after vitamin D administration. Then, the sera were separated by centrifugation and transferred to a -70°C freezer until IL-1 was measured. The inclusion criteria were stroke patients aged 18 to 65, with NIHSS scores between 5 and 15, and vitamin D levels less than 30 ng/mL. Exclusion criteria were hypercalcemia (Ca > 10.5 mg/dL), taking corticosteroids, NSAIDs, statins, a history of cancer, autoimmune disorders, liver diseases, pregnancy, breastfeeding, and vitamin D allergy. Another exclusion criterion was the patient's death within 72 hours of entering the study.

### 3.1. Statistical Analysis

Data were analyzed according to the initial group allocation (intention to treat). The continuous variables' normality was assessed using the Shapiro-Wilk test and Q-Q plot. Mean and standard deviation (SD) were used for reporting continuous variables, and frequency and percentage were used to describe categorical variables. To compare the means of continuous variables between the vitamin D and control groups, parametric or non-parametric tests such as the Student's *t*-test or Mann-Whitney U test were used. The chi-square or Fisher's exact test was used to compare the differences between the frequencies of categorical variables. For comparing the paired continuous values (before and after the intervention), either the Paired *t*-test or Wilcoxon's test was used, depending on the normality of the continuous variables. A linear generalized estimating equations (GEE) model with an independent correlation structure was used to analyze the repeated measurements and assess the changes in NLR, PLR, and NIHSS over seven visits during the study. Also, crude and confounder-adjusted linear GEE with an independent correlation structure was used to assess the effect of the intervention on the mean changes of NLR, PLR, and NIHSS. All statistical analyses were performed at a significance level of less than 0.05 (95% confidence interval (CI)) using STATA software version 14.

## 4. Results

The mean age of the patients was 61.45 ± 4.74 years. There were 18 female (42.86%) and 24 male patients (57.14%). The groups had no significant differences at baseline (Table 1).

**Table 1.** Comparison of General and Clinical Information of Patients Between Vitamin D Recipients and Controls ^a^

Variables	Vitamin D (n = 23, 54.76%)	Control Group (n = 19, 45.24%)	Total (n = 42, 100%)	P-Value
**General information**				
**Age, y**	61.47 ± 4.54	61.42 ± 5.10	61.45 ± 4.74	0.807
**Gender **				0.073
Female	7 (30.43)	11 (57.89)	18 (42.86)	
Male	16 (69.57)	8 (42.11)	24 (57.14)	
**Weight, kg**	74.21 ± 9.70	70.78 ± 10.96	72.66 ± 10.31	0.289
**Height, cm**	172.30 ± 6.60	166.94 ± 7.20	169.88 ± 7.31	0.016 ^b^
**Body mass index (BMI, kg/m** ^**2**^ **)**	24.95 ± 2.61	25.40 ± 3.75	25.16 ± 3.14	0.653
**Length of hospital stay, d**	8.86 ± 7.67	19.57 ± 32.16	13.71 ± 22.69	0.198
**Length of ICU stay, d**	3.26 ± 7.02	6.21 ± 9.81	4.59 ± 8.42	0.750
**Initial vitamin D concentration**	22.12 ± 6.11	21.18 ± 8.18	21.70 ± 7.04	0.989
**Initial APACHE II score **	10.04 ± 1.94	9.78 ± 1.84	9.92 ± 1.87	0.668
**Initial NIHSS score**	11.69 ± 1.60	11.52 ± 1.61	11.61 ± 1.59	0.736
**Biomarkers **				
Initial interleukin-1 (IL-1)	37.00 ± 16.63	9.09 ± 5.33	24.84 ± 8.57	0.350
Interleukin-1 (IL-1) (3 days after intervention)	13.40 ± 20.92	25.05 ± 60.69	18.48 ± 42.74	0.496
Initial interleukin-6 (IL-6)	32.53 ± 61.43	34.82 ± 55.62	33.57 ± 58.18	0.919
Interleukin-6 (IL-6) (3 days after intervention)	26.91 ± 26.61	26.60 ± 25.28	26.77 ± 25.70	0.742
Outcomes				
In-hospital mortality				0.707
No	19 (82.61)	14 (73.68)	33 (78.57)	
Yes	4 (17.39)	5 (26.32)	9 (21.43)	
Intubatio, mean ± SD	1.47 ± 5.02	11.78 ± 32.91	6.14 ± 22.71	0.065
Intubation				
No	20 (86.96)	12 (63.16)	32 (76.19)	0.143
Yes	3 (13.04)	7 (36.84)	10 (23.81)	
**Barthel index (3 months post-discharge)**	13.21 ± 7.48	11.73 ± 8.35	12.54 ± 7.82	0.502

^a^ Values described as mean ± standard deviation or No. (%).

^b^ Statistically significant at P-value < 0.05

According to Table 2, the mean IL-1 decreased compared to before the intervention in the vitamin D group (-23.60 ± 103.83), although this decrease was not statistically significant (P = 0.070). However, in the control group, a significant increase was observed in the mean IL-1 compared to before the intervention (15.96 ± 59.64, P-value = 0.012). In addition, the changes in IL-1 after the intervention were statistically different between the two groups (mean difference of -23.60 ± 103.83 in the vitamin D group vs. 15.96 ± 9.64 in the control group, P-value = 0.002). The mean IL-6 decreased in both groups after the intervention compared to before, although these changes were not statistically significant (P-value > 0.05). Also, the changes in IL-6 after the interventions were not significantly different between the two groups (P-value = 0.704) (Table 2).

**Table 2.** Changes in Interleukin-1 and Interleukin-6 Before and After Intervention in Study Groups ^a^

Biomarkers	Mean Difference ± Standard Deviation Difference	Paired Comparison, P-Value ^b^	Between-Group Comparison ^c^ for IL-1	Between-Group Comparison ^c^ for IL-6
**Vitamin D group**			0.002 ^d^	0.704
Difference of IL-1 ^e^	-23.60 ± 103.83	0.070		
Difference of IL-6	-5.62 ± 53.21	0.493		
**Control group**				
Difference of IL-1 ^e^	15.96 ± 59.64	0.012 ^d^		
Difference of IL-6	-8.22 ± 38.46	0.732		

^a^ Values are mean ± standard deviation

^b^ Comparison of pre and post-intervention values (paired comparison)

^c^ Comparison of differences in interleukin-1 or interleukin-6 between two groups of intervention

^d^ Statistically significant at P-value < 0.05

^e^ Difference = Value after – Value before

Based on the linear GEE results, only NLR in the vitamin D group was significantly increased (P-value_time effect_ = 0.007). Also, there was a significant interaction effect between time and intervention groups in the NLR factor (P-value_time×groups_ = 0.002) (Table 3).

**Table 3.** Changes in NLR, PLR, and NIHSS During the Study ^a^

Factors and Groups	Visit 1	Visit 2	Visit 3	Visit 4	Visit 5	Visit 6	Visit 7	P-Value _Time Effect_	P-Value _Time × Groups_	Comparison of Time Groups ^b^
**NLR**									0.002 ^c^	
Vitamin D	4.68 ± 6.07	5.11 ± 6.26	4.40 ± 4.12	6.19 ± 5.20	6.24 ± 4.21	6.41 ± 5.29	6.69 ± 5.45	0.007 ^c^		Non-significant
Control group	6.86 ± 4.24	6.56 ± 5.74	6.91 ± 4.42	6.14 ± 5.42	6.30 ± 4.44	7.63 ± 4.85	7.10 ± 5.00	0.817		Non-significant
Total	5.66 ± 5.37	5.77 ± 6.00	5.54 ± 4.39	6.16 ± 5.23	6.27 ± 4.26	6.94 ± 5.03	6.92 ± 5.05	0.760	-	Non-significant
**PLR**									0.049 ^c^	
Vitamin D	138.34 ± 113.78	143.69 ± 121.52	128.05 ± 90.67	177.98 ± 143.48	172.20 ± 130.65	202.07 ± 202.15	171.25 ± 65.28	0.059		T1/T5 ^c^
Control group	180.24 ± 81.26	152.21 ± 73.57	164.68 ± 48.35	156.27 ± 81.35	156.75 ± 73.41	151.10 ± 68.26	160.50 ± 103.32	0.620		Non-significant
Total	157.3 ± 101.44	147.54 ± 101.58	144.62 ± 76.01	167.96 ± 117.97	164.47 ± 104.54	179.91 ± 157.68	165.27 ± 86.23	0.509	-	Non-significant
**NIHSS**									< 0.001 ^c^	
Vitamin D	11.69 ± 1.60	11.17 ± 1.80	10.60 ± 2.08	9.66 ± 2.35	9.50 ± 2.52	9.07 ± 2.81	8.62 ± 2.97	< 0.001 ^c^		T1/T2 ^c^, T1/T3 ^c^, T1/T4 ^c^, T1/T5 ^c^, T1/T6 ^c^, T1/T7 ^c^
Control group	11.52 ± 1.61	11.52 ± 1.67	11.31 ± 1.88	10.88 ± 1.96	10.68 ± 2.27	11.00 ± 1.94	11.00 ± 1.94	0.018 ^c^		T1/T4 ^c^, T1/T5 ^c^
Total	11.61 ± 1.59	11.33 ± 1.73	10.92 ± 2.00	10.23 ± 2.24	10.09 ± 2.44	9.91 ± 2.60	9.94 ± 2.66	< 0.001 ^c^	-	T1/T2 ^c^, T1/T3 ^c^, T1/T4 ^c^, T1/T5 ^c^, T1/T6 ^c^, T1/T7 ^c^

^a^ Values are expressed as mean ± standard deviation

^b^ Each visit time compared with visit time

^c^ Statistically significant at P-value < 0.05 based on the generalized estimation equation (GEE) method

Changes in PLR were not statistically significant in vitamin D (P-value_time effect_ = 0.059) and control groups (P-value_time effect_ = 0.620) (Table 3).

The NIHSS score significantly decreased in both groups over the intervention period (P-value_time effect_ in vitamin D group < 0.001 and P-value_time effect_ in control group = 0.018)

**Figure 2.**
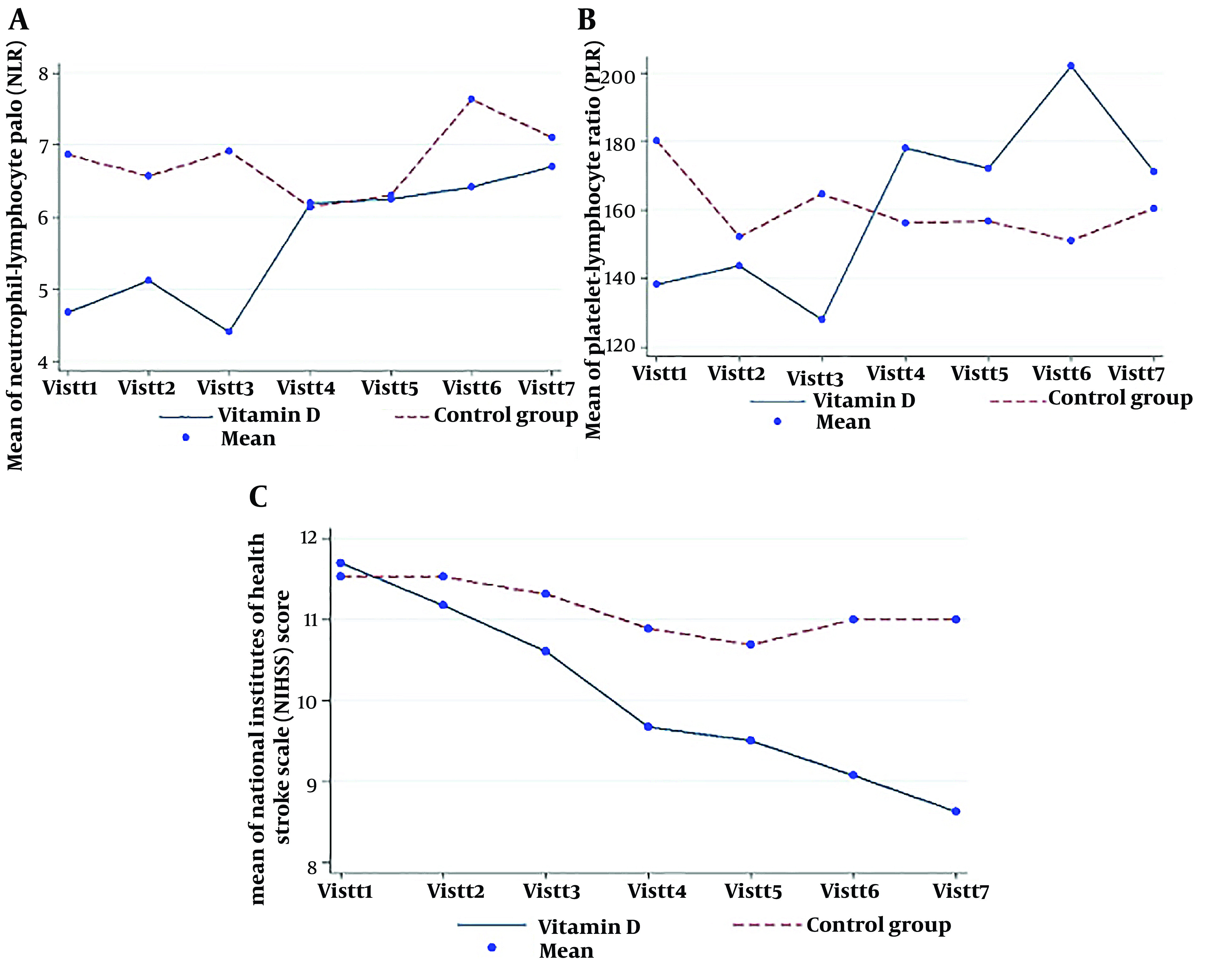
Changes in the mean NLR, PLR, and NIHSS between groups during the study

In addition, there was a significant interaction effect between time and intervention groups in the NIHSS score (P-value_time×groups_ < 0.001) (Table 3 and Figure 2).

**Table 4.** Results of Univariate and Multivariable Linear Generalized Estimating Equation on Effect of Vitamin D on Mean Changes of NLR, PLR, and NIHSS During Study ^a^

Factors and Groups	Model 1: Crude β ^b^, 95% CI	P-Value	Model 2: Adjusted β, 95% CI	P-Value
**NLR**				0.131
Control group	Reference	0.321	Reference	
Vitamin D	-1.25 (-3.72, 1.22)		-1.68 (-3.87, 0.50)	
**PLR**				0.608
Control group	Reference	0.876	Reference	
Vitamin D	-4.23 (-57.28, 48.80)		-9.77 (-47.13, 27.58)	
**NIHSS**				0.530
Control group	Reference	0.145	Reference	
Vitamin D	-0.84 (-1.97, 0.291)		-0.38 (-1.60, 0.82)	

^a^ Model 1: Intercept groups; Model 2: Intercept, gender, age, groups, BMI, initial vitamin D concentration, initial APACHE II score, and length of hospital stay

^b^ Coefficient (β), 95% confidence interval.

Table 4 shows the effect of the intervention on changes in NLR, PLR, and NIHSS. At the univariate level, no association existed between the groups under study and the mean changes in the desired factors. After taking into account confounding factors such as age, gender, body mass index, the amount of vitamin D before the intervention, the Apache score, and the duration of hospitalization, there was no statistically significant association between the type of interventions and the mean changes in the desired factors (P-value > 0.05). Although, in the group receiving vitamin D compared to the control group, the mean NLR decreased by about 2 units (β = -1.68, 95%CI: -3.87, 0.50), the PLR value decreased by about 10 units (β = -9.77, 95%CI: -47.13, 27.58), and the mean NIHSS score decreased by about one unit during the study (β = -0.38, 95%CI: -1.60, 0.82) (P-value > 0.05).

## 5. Discussion

We know the neuroprotective effects of vitamin D in stroke patients and its anti-inflammatory effects, but there are not enough clinical trials on the effect of this vitamin on pro-inflammatory and inflammatory cytokines, especially IL-1. In addition, its administration dose and duration are not yet known. Thus, we decided to determine the effect of prescribing a high dose of vitamin D on anti-inflammatory parameters short-term and long-term prognosis of patients with ischemic stroke. In the vitamin D group, the mean IL-1 decreased compared to before the intervention; although this decrease was not statistically significant, it was borderline. In the control group, a significant increase was observed in the mean IL-1 compared to before the intervention.

Regarding the IL-6 factor in both groups after the intervention compared to before, the mean IL-6 decreased, although these changes were not statistically significant. No association existed between the groups under study and the mean changes in NLR, PLR, and NIHSS. Although, in the group receiving vitamin D compared to the control group, the mean NLR decreased by about 2 units, the PLR value decreased by about 10 units, and the NIHSS score decreased by about one unit during the study. These decreases were clinically important but not statistically significant because safe interventions like vitamin D can improve the patient's status.

Kadri et al. determined the effect of vitamin A and D combined supplements on interleukin-1β (IL-1β) and clinical outcome in a randomized clinical trial conducted on 120 ischemic stroke patients in four groups (vitamin A or D only, combined vitamin A and D, and placebo for 12 weeks). The combination group showed significant increments in vitamin A and D serum levels after 12 weeks of treatment compared to the other groups. In contrast, IL-1β serum level and NIHSS score significantly decreased in the combination group. Combined vitamin A and D significantly increased vitamin A and D serum levels, decreased IL-1β serum levels, and ultimately improved clinical outcomes in ischemic stroke patients (22).

Like our study, Kadri et al. (22) reported the effect of vitamin D on the recovery process of stroke patients. In a study by Rezaei conducted on 60 patients, the first group received a single dose of 300,000 units of intramuscular vitamin D, and the second group did not receive vitamin D supplementation. The level of interleukin 6 significantly decreased after treatment with vitamin D (23).

Narasimhan and Balasubramanian conducted a study on 60 patients divided into two groups of 30 people. The first group received a single intramuscular dose of cholecalciferol 6 lac I.U/m, and the second group did not receive vitamin D. The prognosis of patients assessed with Scandinavian criteria Stroke Scale (SSS) was significantly higher in the group receiving vitamin D (24). In another study by Qiu et al., the vitamin D level in stroke patients had a significant negative relationship with the mortality rate [OR, 0.72; P < 0.001] and stroke recurrence [OR, 0.77; P < 0.001] (25).

Besides, in a study by Wang et al., which was conducted on stroke patients, the vitamin D level had a negative relationship with inflammatory factors, including interleukin 6 and C-reactive protein, showing the importance and anti-inflammatory properties of vitamin D in the treatment of patients with acute stroke (26). Zittermann et al. revealed that treatment with vitamin D statistically decreased TNF-α. In this study, treatment with oral vitamin D, equivalent to 3,332 IU of cholecalciferol daily, reduced the level of TNF-α by 10% (27). We discovered that high-dose vitamin D is unnecessary and cannot be more effective. When writing this manuscript, we are amid the COVID-19 pandemic. As known, COVID-19 is a complex disease that affects multiple organs in patients, such as the kidneys, lungs, eyes, sense of taste, liver, and brain (28-34). As patients are predisposed to coagulation disorder, the risk of stroke can increase (35). Therefore, it is important to pay attention to this disease, its complications, and timely vaccination to prevent and even treat stroke (36).

### 5.1. Conclusions

According to the results, a high dose of vitamin D can improve the National Institute of Health Stroke Scale and decrease interleukin-1 and interleukin-6 levels, although these changes were not statistically significant. The mean neutrophil-to-lymphocyte ratio and platelet-to-lymphocyte ratio decreased after using high-dose vitamin D.

## Data Availability

The dataset presented in the study is available on request from the corresponding author during submission or after publication.

## Clinical Trial Registration Code:

IRCT20200213046484N2
